# Implementierung des* Objective Structured Clinical Examination* (OSCE) im Masterstudiengang Pflegewissenschaft – Pilotprojekt zur Prüfung der ethischen Kompetenz in Pflegepraxis und -forschung

**DOI:** 10.1007/s00481-022-00701-1

**Published:** 2022-04-26

**Authors:** Christine Dunger, Martin W. Schnell

**Affiliations:** Lehrstuhl für Sozialphilosophie und Ethik im Gesundheitswesen, Fakultät für Medizin, Private Universität Witten/Herdecke gGmbH, Alfred-Herrhausen-Straße 50, 58448 Witten, Deutschland

**Keywords:** Objective Structured Clinical Examination (OSCE), Postgraduelle Lehre, Ethische Kompetenzen, Pflegewissenschaft, Prüfformat, Objective Structured Clinical Examination (OSCE), Postgraduate education, Ethical competencies, Nursing science, Test format

## Abstract

Die Vermittlung ethischer Kompetenzen ist wesentlicher Bestandteil in berufsqualifizierenden und postgraduellen Studiengängen. Dabei werden praktisch-ethische Problemlösungskompetenzen, je nach Studiengang aber auch die forschungsethische Betrachtung von Studien thematisiert. Die Überprüfung dieser ethischen Kompetenzen stellt sich als Herausforderung dar. Das schriftliche oder mündliche Abfragen von Lehrinhalten greift zu kurz, da somit lediglich Wissen, jedoch nicht Fertigkeiten oder gar Haltung erfasst werden können.

Bei der Reakkreditierung des Masterstudiengangs Pflegewissenschaft an der Universität Witten/Herdecke wurde dieser Herausforderung mit der Umsetzung eines für die postgraduelle Ausbildung innovativen Prüfungsformats begegnet. Für das Modul „Ethik in der Pflegepraxis und -forschung“ wurde ein Format ausgearbeitet, welches sich an den „Objective Structured Clinical Examinations“ orientiert und somit die erworbenen Fertigkeiten und Haltungen der Studierenden mit einbezieht. Der Beitrag zeigt auf, wie das OSCE-Prüfungsformat (kurz: OSCE) konzipiert und eine Adaption erfolgt ist.

## Abstract (en)

**Background:**

Teaching ethical competencies is an essential component of professional and postgraduate curricula. Developing practical–ethical problem-solving competencies as well as appraising program-specific studies and related research ethics are topics typically addressed. However, assessment of these ethical competencies poses a challenge. Written or oral assessment formats addressing relevant learning objectives is mainly limited to knowledge testing alone, often not capturing relevant skills or attitudes pertinent to those competencies.

**Aim:**

During the reaccreditation of the masters of science (MSc) program in Nursing Science at Witten/Herdecke University, this challenge was addressed by implementation of an innovative examination format for postgraduate education.

**Procedure:**

For the module “Ethics in nursing practice and research” a format based on the Objective Structured Clinical Examination (OSCE) format was developed. This assessment method incorporates evaluation of acquired skills and attitudes of nursing students.

**Conclusion:**

The article demonstrates development and adaption of the OSCE format for this purpose.

## Hintergrund und Ziel

### OSCE – ein strukturiertes Praxisprüfungsformat

Das *Objective Structured Clinical Examination-Prüfformat*, im Folgenden kurz OSCE genannt, ist eine vorstrukturierte Prüfungsform zur objektiven Beurteilung klinisch-praktischer Fertigkeiten. Zunächst in der Ausbildung von Humanmediziner:innen etabliert, werden OSCEs heute international ebenso in der Ausbildung weiterer Gesundheitsberufe angewandt. Sie orientieren sich an festen Gütekriterien, welche die Reliabilität und Validität des Prüfungsformates sicherstellen sollen (vgl. Schultz et al. 2008; König et al. 2002; Chenot und Ehrhardt 2003).

Ein OSCE besteht aus mehreren Prüfungsstationen mit unterschiedlichen Aufgabenstellungen, welche Wissen, aber auch praktische Fertigkeiten und Haltungen beobachtbar machen sollen (Jäger et al. 2008). Die Praxistauglichkeit der Situationen und ihre Standardisierung spielen dabei eine große Rolle (Schultz et al. 2008). Gleiches gilt für die Bewertungsinstrumente, die immer wieder auf Testgütekriterien untersucht werden (Schultz et al. 2008; Nikendei und Jünger 2006). Sie können aus Checklisten mit einfachen Bewertungen (erfüllt/nicht erfüllt), aber auch mehrstufigen Bewertungen bestehen. Hinzu kommen globale Ratings, die insbesondere zur Einschätzung „weicher Faktoren“ wie Zugewandtheit oder Kommunikation genutzt werden. Diese zeigen jedoch nicht dieselbe Güte wie die Erfassung klinischer Fertigkeiten (Brannick et al. 2011). Je nach Fokus können die Prüfungssituationen und -bewertungen zudem unterschiedliche Schwerpunkte haben. Prüfungssituationen mit Fokus auf das erlangte Wissen bauen vornehmlich auf Seminar- und Vorlesungsinhalte auf und können standardisiert bewertet werden. Die kritische Einschätzung von Fallgeschichten kann hingegen vor allem in der Reflexion von Übungsaufgaben thematisiert werden und entzieht sich einer klassisch-standardisierten Bewertungslogik. Hier ist vor allem darauf zu achten, dass es mehrere Prüfer gibt, deren teilweise subjektive Sichtweisen sich ausgleichen (Chenot und Ehrhardt 2003).

Je Prüfungsdurchlauf und -gruppe sind somit mehrere Stationen zu bewältigen, die auf verschiedene prüfungsrelevante Inhalte fokussieren. Hierzu können auch Schauspieler:innen eingesetzt werden, die an der Simulation teilnehmen und, je nach Ausgestaltung, an der Bewertung beteiligt sind. Obgleich die Prüfungsinhalte gleichbleiben, werden bei einer neuen Prüfungsgruppe die Situationen und Aufgaben abgeändert. Auch deshalb ist eine gute „Konstruktion“ der Aufgaben wichtig. Jeffrey et al. (2014) schlagen hierfür die Verwendung von „Best-practice“-Leitlinien vor.

Auch für die formale Planung, d. h. die Dauer und Anzahl der Stationen in dem Prüfungsparcours gibt es Empfehlungen (Nikendei und Jünger 2006). Generell gilt, dass die Dauer der einzelnen Prüfungssituationen geringer werden sollte, je mehr Stationen zu bewältigen sind. Unbedingt einzuhalten sind kurze Pausen, die es den Studierenden ermöglichen, sich zu entspannen und auf die neue Situation einzustellen. Die Bewertung kann formativ oder summativ und kompensatorisch oder non-kompensatorisch durchgeführt werden (Nikendei und Jünger 2006). Tab. 1 gibt kurze Definitionen dazu. Das Feedback im Rahmen formativer Prüfungen erfolgt zumeist mündlich (*face-to-face*) und im Anschluss an die Prüfungssituation. Es sollte von den Prüfer:innen trainiert werden (Perron et al. 2016). Zunehmend werden jedoch auch Konzepte diskutiert und Prüfungsformate angewendet, in denen ein Feedback im Rahmen summativer Prüfungen aufgegriffen wird. Dieses kann ebenfalls mündlich (*face-to-face*), schriftlich oder als aufgenommenes elektronisches Feedback gegeben werden (Harrison et al. 2015; Sterz et al. 2021).

**Table 1:** 

Prüfungsart	Bedeutung
Formativ	Kurze, direkte Feedbackschleifen werden in den Prüfungsverlauf einbezogen
Summativ	Während der Prüfung findet kein Feedback statt
Kompensatorisch	Eine Kompensation schlechterer Teilergebnisse durch bessere Ergebnisse in anderen Prüfungsbereichen ist möglich
Non-kompensatorisch	Eine Kompensation ist nicht möglich

Neben dieser Einteilung sind verschiedene Formen der Lernfortschrittskontrolle und Prüfungsformen zu berücksichtigen. Zum einen lassen sich auch hier formative und summative Assessments unterscheiden. Diese werden verstanden als fördernde, das Lernen begünstigende und selektierende, das Lernen prüfende Verfahren. Hinzu kommen diagnostische Assessments, die ebenfalls fordernden (Self-Assessment) oder selektierenden (Eignungstest) Charakter haben können (Ehlers et al. 2013). Die jeweils abzuprüfenden Kompetenzen zeigt Millers Kompetenzpyramide auf, welche zwischen den vier Stufen „knows“, „knows how“, „shows how“ und „does“ differenziert (Chenot und Ehrhardt 2003). Das hier vorgestellte OSCE-Prüfungsformat ist als Modulabschlussprüfung als summatives Assessment zu verstehen, welches eine für die Praxis relevante Leistung mittels standardisierter Darsteller:innen prüft.

### Prüfungen praktisch-ethischer Kompetenzen in Pflegepraxis und -wissenschaft

Auch in der grundständigen Ausbildung von Pflegenden (Schlegel 2018) und in der postgraduellen Ausbildung (Jeffrey et al. 2014; MacDonald-Wicks und Levett-Jones 2012) werden OSCEs angewendet. Beyer et al. (2016) zeigen in einem Review zu pflegewissenschaftlichen Publikationen zu OSCEs und den darin beschriebenen Kompetenzen, dass international, mit Schwerpunkt auf Großbritannien, Kanada und Australien, unterschiedlichste pflegerische Kompetenzen gemessen werden. Insbesondere konkrete fachliche Fertigkeiten, die gut beobachtet und bewertet werden können, aber auch Wissen und Haltung werden getestet. Dabei ist die Möglichkeit der Kombination von Kompetenzen in einer Prüfungssituation besonders zu berücksichtigen. Zur Bewältigung einer Aufgabenstellung müssen die Studierenden auf ihr Wissen zurückgreifen, dieses anwenden und vermitteln können sowie, bei entsprechender Formulierung, ihre eigene Einschätzung argumentativ vortragen können.

In berufsqualifizierenden Studiengängen bietet dies die Möglichkeit der Prüfung situativ eingebetteter, komplexer Situationen, die den zu bewältigenden pflegepraktischen Versorgungssituationen zwar nicht vollständig entsprechen, sie jedoch simulieren. Diese Simulation ist auch auf postgraduellem Niveau von Vorteil, wenn erworbenes Wissen, Fertigkeiten und Haltung geprüft werden sollen. Diese beziehen sich in der postgraduellen Ausbildung jedoch auf Kompetenzen in Forschung und erweiterter Praxis. OSCEs zeigen somit auch in der Prüfung praktisch-ethischer Kompetenzen in der Pflegepraxis und -wissenschaft ihr besonderes Potenzial. Da sie bisher jedoch in der Pflege weder in dieser Ausrichtung noch auf akademischem Niveau durchgeführt werden, sind sie als Prüfungsformat grundsätzlich anzupassen.

### Ziel

Ziel des Beitrages ist darzustellen, wie das OSCE-Prüfungsformat für die postgraduelle Lehre im Bereich Ethik genutzt werden kann. Am Beispiel des Masterstudiengangs Pflegewissenschaft an der Universität Witten/Herdecke wird eine entsprechende Konzeption und Adaption beschrieben.

## Vorgehen zur Adaption des OSCE-Prüfungsformates für das Modul Ethik in Pflegepraxis und -wissenschaft

**Figure 1:**
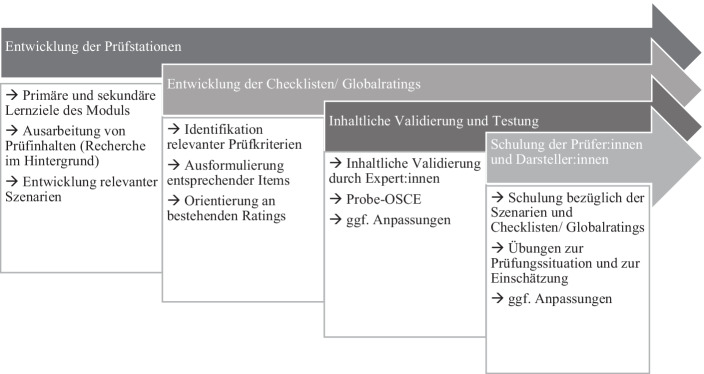

Das hier beschriebene Vorgehen greift zunächst die inhaltliche Passung zwischen Prüfungsform und Seminarinhalten auf, um dann die formalen und inhaltlichen Entwicklungsschritte aufzuzeigen. Die Entwicklung der Prüfungsstationen ist in Abb. 1 prozesshaft dargestellt.

### Passung des OSCE-Prüfungsformates mit Seminarinhalten und -didaktik

Wie bereits beschrieben, soll der durchzuführende „Ethik-OSCE“ Wissen, Fertigkeiten und Haltung der Studierenden erfassen, die das Modul „Ethik in der Pflegepraxis und -forschung“ absolviert haben. Der Fokus liegt dabei explizit auf den Kompetenzen, die in der anvisierten Berufsausübung nach dem Masterabschluss von den Studierenden gefordert werden. Dazu gehören aus Sicht des Moduls bspw., dass die Studierenden die in der Medizin, aber auch in demokratischen Staaten relevanten Basiswerte Autonomie, Fürsorge und Gerechtigkeit (Schnell 2017) kennen und sie im Kontext professioneller Pflege diskutieren können. Ebenso sollen sie notwendige Maßnahmen auf den verschiedenen Handlungsebenen in ethischer Hinsicht reflektieren können (siehe „Inhaltliche Entwicklung der Prüfungssituation und Checklisten“).

Die Lernergebnisse können mittels OSCE geprüft werden, was den didaktischen, konstruktivistisch und konnektivistisch geprägten Lehrmethoden, mittels derer sie im Seminar eingeübt werden, entspricht. Damit folgt die Entwicklung des „Ethik-OSCEs“ den Anforderungen des *Constructive Alignment* (Biggs und Tang 2011), in dessen Mittelpunkt als didaktischem Konzept ebendiese Abstimmung steht.

Das Modul bietet somit zahlreiche Ansatzpunkte, um innerhalb der Seminare inhaltlich die verschiedenen Prüfungsstationen vorzubereiten und das Prüfungsformat didaktisch einzuleiten. Inhaltlich lassen sich verschiedene Themenblöcke identifizieren, die für mögliche Prüfungsstationen geeignet sind. Dazu gehören klinisch-ethische Fallbesprechungen zu verschiedenen Konfliktsituationen, ebenso wie die Anforderungen an gelebte wissenschaftliche Integrität im Sinne einer guten wissenschaftlichen Praxis. Didaktisch ermöglichen Seminare, diskursive Unterrichtsformate und die Bearbeitung klinisch-ethisch herausfordernder Fallbeispiele sowie organisationsethischer Fragen, das erworbene Wissen anzuwenden und Fertigkeiten für die Problemlösung einzuüben. Die Vertiefung von forschungsethischer Reflexion und Einübung von Verhaltensweisen im Forschungsprozess werden ebenfalls durch praktische Übungen und Peer-feedback gefördert.

### Formale Aspekte des Prüfungsformates

Angesichts der möglichen Ausgestaltung eines OSCEs sind zu Beginn der Entwicklung einige Grundentscheidungen zu treffen. In der Entwicklung des Prüfungsparcours ist beispielsweise zu berücksichtigen, dass die genannten Prüfungssituationen sehr komplex sind. Sie erfordern ein hohes Maß an Konzentration und ein Sich-Einlassen auf das jeweilige Szenario. Ebenfalls zu berücksichtigen ist, dass ethische Kompetenzen im Rahmen praktisch-ethischer Herausforderungen und im Sinne forschungsethischen Verhaltens geprüft werden und gleichermaßen vertreten sein sollen. Das erfordert ebenfalls eine gedankliche Umorientierung der Studierenden. Daher werden nur wenige Prüfungsstationen eingeplant, so dass die Studierenden ausreichend Zeit zur Vorbereitung sowie eine kurze Pause nach jeder Station haben. Zudem gibt es keine direkten Feedbacks, um mögliche Unsicherheiten im Prüfungsverlauf zu vermeiden. Die somit summativ angelegte Prüfung wird jedoch in einem Gesamtfeedback nachbereitet.

Neben den genannten Kriterien zur Bewältigung der Prüfungssituation für die Studierenden sind bei der Entwicklung auch Ressourcenaufwand und Kosten im Blick zu halten (Rau et al. 2011; Chenot und Ehrhardt 2003). Auch deswegen wird die Bearbeitungszeit der Prüfungsstationen begrenzt. Für das „Ethik-OSCE“-Prüfungsformat wird festgelegt, dass nach einer 3‑minütigen Vorbereitung, eine 5‑minütige Prüfung erfolgt, an die sich wiederum eine 2‑minütige Erholungspause anschließt. Für jede/n Studierende/n ergibt sich so bei vier Prüfungsstationen eine 40-minütige Prüfung. Die Zeitintervalle wurden in einem Probe-OSCE überprüft und danach festgelegt.

Für die gesamte Prüfungszeit wirken acht Personen als Interaktionspartner:innen und Prüfer:innen an der Prüfung mit. Die Schulung dieser Interaktionspartner:innen und Prüfer:innen ist ein wichtiger letzter Schritt. Da die Prüfungssituationen standardisiert sind, werden die Interaktionspartner:innen so angeleitet, dass sie die jeweilige Situation entlang eines Skripts gestalten. Diese Skripte orientieren sich wiederum an in den Seminaren eingeübten Inhalten. Die Prüfer:innen sind Lehrende am Department für Pflegewissenschaft und am Department für Humanmedizin der Universität Witten/Herdecke, die mindestens einen Masterabschluss haben. Auch sie erhalten eine Einführung in das Prüfungsformat OSCE und in die Bewertungsinstrumente.

Zur Bewertung werden standardisierte Checklisten und globale Ratings eingesetzt, die eine Schulung der beteiligten Prüfer:innen erfordern. Neben den beobachtenden, nicht-teilnehmenden Prüfer:innen sind auch die Interaktionspartner:innen im Rahmen der Prüfungssituation beteiligt. Sie nehmen an dem globalen Rating teil, um auch die erlebte verbale und nonverbale Kommunikation einzubeziehen. Nach der Prüfung wird eine Gesamtnote festgelegt, wodurch eine Kompensation schlechterer Teilergebnisse durch bessere möglich ist.

Bei größeren Kohorten ergeben sich mehrere Prüfungsgruppen, für die der Prüfungsparcours jeweils inhaltlich verändert werden muss. In diesem Fall ist eine Orientierung an formalen Vorgaben – im Sinne einer „best practice“ –, die in den Seminaren vermittelt werden, umso wichtiger. Das ist in der Konzeption insofern berücksichtigt, als die Prüfungssituationen durch Expert:innen validiert wurden und in entsprechenden Variationen vorliegen.

### Inhaltliche Entwicklung der Prüfungssituationen und Checklisten

Wie beschrieben, erfolgt die inhaltliche Entwicklung der Prüfungssituationen, d. h. der OSCE-Stationen in Übereinstimmung mit den Lernzielen und Seminarinhalten des Moduls. Diese Lernziele sind, dass die Studierenden

- um die Definition von Ethik und deren Bedeutung im pflegerischen Kontext wissen
- die relevanten Basisethiken kennen und diese kritisch diskutieren können
- die demokratischen Basiswerte Autonomie, Fürsorge und Gerechtigkeit im Kontext professioneller Pflege diskutieren können
- die Basiswerte in der Diskussion ethisch relevanter Herausforderungen nutzen
- notwendige Maßnahmen auf verschiedenen Handlungsebenen auch in ethischer Hinsicht reflektieren und begründen
- ein detailliertes Bild vom ethisch verantwortlich tätigen Wissenschaftler entwickeln und damit verbundene Anforderungen in eigenen Projekten umsetzen können

Das im Folgenden aufgegriffene Beispiel nimmt Bezug zu den Lernzielen an dritter bis fünfter Stelle. Wie diese vermittelt werden, um sie im Rahmen einer praktischen Prüfung testen zu können, wird kurz anhand entsprechender Seminarinhalte und deren didaktischer Aufbereitung aufgegriffen.

So entstehen entlang der Kompetenzen (Wissen, Fertigkeiten und Haltung) verschiedene Gesprächs‑, Beratungs- und Entscheidungsanlässe, die in den Prüfungssituationen dargestellt werden. Für jeden dieser Anlässe gibt es wiederum verschiedene Szenarien, um die Situation variieren zu können.

Die Anlässe, die in der Aufgabenstellung ausformuliert und erläutert werden, lauten bspw. „Gültigkeit und Nutzen ethischer Fallbesprechungen“ oder „Teilnehmernachfrage zu Unterlagen zum Informed Consent“. In die Erarbeitung der Szenarien werden existierende Leitlinien oder Leitfäden einbezogen. Es findet zudem eine inhaltliche Validierung der Szenarien durch Expert:innen statt, in deren Anschluss die angemerkten Veränderungen umgesetzt werden.

Die Entwicklung der Checklisten und Globalratings verläuft parallel zur Entwicklung der Prüfungsstationen. Die Checklisten orientieren sich an den inhaltlichen und formalen Kriterien der jeweiligen Anlässe, die die Entwicklung relevanter Prüfkriterien erlauben. Insgesamt sind sowohl binäre Items (z. B. Der Student/die Studentin hält Augenkontakt: ja/nein) als auch Likert Skalen (siehe Tab. 2) enthalten. Das Globalrating orientiert sich an dem *Berliner Global Rating Instrument* (BGR) (Scheffer 2009), welches übersetzt und speziell für OSCE-Prüfungen mit Studierenden-Simulationspatient:innen-Interaktionen entwickelt sowie getestet wurde. Das Globalrating wird von Interaktionspartner:innen und Prüfer:innen durchgeführt.

**Table 2:** 

		Nicht umgesetzt		Vollständig umgesetzt		
	Der Student/die Studentin	0	1	2	3	Hinweise für die Prüfer:innen
1	… sagt, was die Aufgaben eines Ethikkomitees sind					Fallbesprechungen, Leitlinienerstellung, Fort‑/Weiterbildungen (Inhaltliche und strukturelle Beratung)
2	… erläutert, inwiefern Beschlüsse nach ethischen Fallbesprechungen bindend sind					Sind sie nicht. Sie müssen jedoch Geltung haben
3	… spricht die verantwortungsvolle Positionen der ausführenden Ärzte an					Die behandelnden Ärzt:innen haben die Therapieverantwortung. Diese Verantwortung und Entscheidungsgewalt wird nicht genommen
4	… verweist auf die Begründungs- bzw. Dokumentationspflicht, falls der Arzt anders handelt, als es der Beschluss nach der Fallbesprechung vorsieht					Eine gegenteilige Entscheidung zur Empfehlung der Kommission muss begründet werden (in der Dokumentation)
5	… erläutert, dass ethische und rechtliche Aspekte zur Realisierung des Patientenwillens immer Vorrang haben					Ethisch und rechtlich stehen Patientenwille und Autonomie an oberster Stelle. Das gilt auch für nichteinwilligungsfähige Patient:innen und wenn der Wille konträr zur ärztlichen Meinung steht
6	… verdeutlicht, dass pragmatisch nicht paternalistisch meinen kann					Pragmatische Lösungen zu finden heißt nicht, dass der Meinung/Einschätzung der therapierenden Ärzt:innen gefolgt wird, weil diese zu wissen glauben, was für Patient:innen das Beste ist. Es meint handlungs-/sachbezogene Entscheidung zu finden

Skalierung: 0 = nicht angesprochen; 1 = erwähnt/angesprochen (nach indirektem Hinweis durch Darsteller:in); 2 = selbständig angesprochen; 3 = selbständig angesprochen und erläutert

Für eine solche Standardisierung spricht die Vergleichbarkeit und Objektivität der Prüfungssituation und Prüfer:innen-Einschätzungen. Eine Bewertung mittels entsprechender Checkliste und globalem Rating erscheint zudem gut möglich und auch sinnvoll, um einerseits eine Standardisierung und andererseits eine Gleichbehandlung der Studierenden zu erreichen (Nikendei und Jünger 2006). Besonders gut operationalisieren lässt sich damit die Bewertung von Wissen/einzubringenden Argumenten und notwendigen Fragen oder Verhaltensweisen, die in den Fallbeispielen angelegt werden. Daneben erscheinen auch einige „weiche Faktoren“ relevant, die über das Globalrating erfasst werden können. Dazu gehören kommunikative Fähigkeiten, aber auch Kritik- und Argumentationsfähigkeit.

Abb. 1 gibt einen Überblick über die Entwicklung der Prüfstationen, die stark inhaltlich geprägt ist, aber auch Bezug zur Validierung und Implementierung nimmt.

## Prüfungssituationen, Checklisten und inhaltlich didaktische Verknüpfung des Prüfungsformates – ein Beispiel

Im Folgenden soll beispielhaft eine Prüfungssituation inklusive der entsprechenden Checklisten und ihrer Verknüpfung mit den Lernzielen, -ergebnissen und -methoden aufgezeigt werden. Gegliedert ist die Darstellung in die entsprechenden Unterkapitel.

### Prüfungssituation und Checklisten

Das hier vorgestellte Szenario bezieht sich auf den Nutzen ethischer Fallbesprechungen, die systematisch validiert werden. Es ist als konflikthafte Interaktion angelegt und enthält zudem Elemente einer mündlichen Kurzprüfung.

Zunächst erfolgt eine Anweisung für die Studierenden bezüglich der Situation, um sich innerhalb der 3‑minütigen Vorbereitungszeit auf die Situation einzustellen.

#### Anweisung für die Studierenden

Situation: Das Ethikkomitee des Krankenhauses berät über den Fall der Behandlung eines Patienten, der eine Patientenverfügung und einen Betreuer hat.

Das Komitee hält in seinem Beschluss fest, dass der Wille des Patienten, der vom Betreuer gemäß der Patientenverfügung zum Ausdruck gebracht wird, maßgeblich für die mögliche Einwilligung in die anstehende Behandlung ist und daher einen Vorrang vor anderen Werten hat.

Ein erfahrener Stationsarzt, dem der Beschluss des Komitees mitgeteilt wird, ist damit nicht einverstanden. Er bittet Sie als leitende Pflegeperson und Teilnehmende an der Beschlussfassung des Komitees daher um ein Gespräch.

##### Aufgabe

Führen Sie ein Gespräch mit dem Arzt, in welchem Sie auf seine Argumente eingehen und zugleich die Position des Ethikkomitees sowie die Bedeutung des Beschlusses verdeutlichen. Sie haben 5 min Zeit.

Neben dieser Aufgabenstellung, die die Studierenden bekommen, erhalten auch die Interaktionspartner:innen und Prüfer:innen jeweils Informationen zu dem Szenario. Für die Darsteller:innen sind darin sowohl Informationen zur Situation, als auch zu ihrer Rolle und ihrem Verhalten in der Interaktion enthalten. Die Prüfer:innen benötigen die Informationen zu den eingebetteten Hinweisen und Informationen ebenfalls, um die Bewertung anhand der jeweiligen Checkliste vorzunehmen.

#### Standardisiertes Skript für den „Arzt“ im Gespräch

Situation: s. oben; identisch mit der Beschreibung für die Studierenden.

Ihre Rolle: Sie sind aufgewühlt und etwas genervt, weil durch die Besprechung alles so fürchterlich kompliziert wird. Zudem finden Sie, dass Sie als ausgebildeter und verantwortlicher Arzt nicht an die Beschlüsse des Ethikkomitees gebunden sind. Diese Position möchten Sie äußern und auch verdeutlichen, dass Sie wissen, was für Ihre Patienten gut bzw. richtig ist. Das wussten Sie schon zu den Zeiten, in denen es noch kein Ethikkomitee gab, welches alle Entscheidungen infrage stellte. Sie sind aber nicht sauer und werden nicht laut, sondern möchten nur Ihre Sichtweise klarstellen.

Ihr Verhalten: Zu Beginn sagen Sie „Ah, gut dass Sie kurz Zeit haben! Ich möchte mit Ihnen kurz über diese Beschlussfassung des Ethikkomitees sprechen. Um nicht lange drumherum zu reden, bin ich damit so nicht einverstanden und möchte das auch ganz klar verdeutlichen. Ich bin der behandelnde Arzt und ausgebildet, um zu wissen, welche Behandlung wann die Beste für meine Patienten ist.“

Im Verlaufe des Gespräches bringen Sie ein,

- dass der behandelnde Arzt am besten wisse, was für den Patienten richtig sei,
- dass man im Alltag pragmatisch vorgehen müsse und daher nicht immer nur ethisch ausgerichtet sein könne,
- dass ein Ethikforum all das gar nicht berücksichtigen könne und ein Beschluss daher auch nicht bindend sei.

#### Situationsspezifische Checkliste für die Prüfer:innen

Die situationsspezifische Checkliste schließlich greift die in dem Szenario angelegten Prüfungsinhalte auf und enthält in der Regel 6 Items. Das hier gegebene Beispiel einer Checkliste verdeutlicht, wie wichtig eine inhaltliche Aufbereitung und Schulung der Prüfer:innen ist. Natürlich können die jeweiligen Informationen, insbesondere zu Wissensinhalten, nochmals gegeben werden, wie die Beschreibung der Bewertungskriterien verdeutlicht. Sie müssen jedoch grundsätzlich präsent sein, damit eine Einschätzung innerhalb der interaktiven Prüfungssituation überhaupt möglich ist.

Die Gesamtnote der Studierenden ergibt sich aus der in allen Prüfungsstationen erreichten Punktzahl (aus Checkliste und Globalrating). Items, die von weniger als 10 % der Studierenden erfüllt wurden, werden nachträglich aus der Checklistenbewertung entfernt. Die Bestehensgrenze wird auf 50 % der Maximalpunktzahl festgesetzt.

### Verknüpfung der Prüfungssituation mit den Lernzielen und Seminarinhalten

Die dargestellte Prüfungssituation entspricht den oben genannten allgemeinen Lernzielen des Moduls, indem sie es ermöglicht zu beurteilen, ob die Studierenden Basiswerte wie Autonomie, Fürsorge und Gerechtigkeit im Kontext professioneller Pflege diskutieren können, und ob sie notwendige Maßnahmen auf der hier angemessenen Handlungsebene in ethischer Hinsicht reflektieren, begründen und diskutieren können. Konkret in der Prüfungssituation bedeutet dies, dass folgende Bewertungen vorgenommen werden:

- Wissen: Weiß der Student/die Studentin, was Aufgabe eines Ethikkomitees und Bindungswirkung eines Beschlusses sind?
- Fertigkeiten: Kann der Student/die Studentin auf den Arzt-Schauspieler eingehen und das Wissen zur Geltung bringen?
- Haltung: Zeigt der Student/die Studentin eine Werteinstellung, die auf den Pragmatismus des Arzt-Schauspielers antwortet?

In den Seminaren werden diese Inhalte anhand von (a) theoretischem Input in Seminarform mit selbständiger Textarbeit zur Nachbereitung, (b) kritischen Diskussionen zur Implementierung sowie Aufgaben und Grenzen klinischer Ethikkomitees in der Praxis und (c) Gruppenübungen vermittelt. Die Gruppenübungen beinhalten in einem ersten Schritt die Simulation einer Fallbesprechung, d. h. die strukturierte Bearbeitung eines Fallbeispiels. Diese Gruppendiskussion und ihre Ergebnisse werden festgehalten und den anderen Arbeitsgruppen als Video, besprochene Präsentation, o. ä. zur Verfügung gestellt. Im zweiten Schritt erfolgen eine gemeinsame Diskussion der Fallbeispiele und eine Reflexion des Vorgehens. So lernen die Studierenden nicht nur das Werkzeug der Fallbesprechung kennen, sondern auch über ethische Konflikte und Herausforderungen kritisch zu diskutieren. Diese Versprachlichung ist für die praktische Prüfung unabdingbar.

## Diskussion und Ausblick

Das in diesem Beitrag vorgestellte Prüfungsformat eines „Ethik-OSCEs“ auf postgraduellem Niveau unter Berücksichtigung praktisch-ethischer, organisationsethischer und forschungsethischer Lehrinhalte in der Pflegewissenschaft ist ein Pilotprojekt. Bislang werden im deutschsprachigen Raum vor allem in Ausbildung und grundständigem Studium, äquivalent zur Entwicklung im Humanmedizinstudium, OSCEs eingesetzt, um klinisch-praktische Fertigkeiten zu überprüfen (Schlegel 2018). Diese OSCEs zur Prüfung klinischer Fertigkeiten sind auch international üblich und vielfach untersucht. So spricht die Studienlage dafür, dass das OSCE-Prüfungsformat insbesondere klinische Kompetenzen auf diesem Kompetenzniveau besser erfassen kann als traditionell klinische Prüfungssituationen (Vincent et al. 2022). Zugleich sind die Umsetzung und Standardisierung des Prüfungsformates wichtige Aspekte, die unmittelbar mit dessen Qualität und Wertigkeit verbunden sind (Goh et al. 2019). Ein neueres Review beschreibt OSCE-Prüfungsformate jedoch auch für den postgraduellen Bereich als wertvolles Lern- und Prüfungselement, wobei die Effekte im Vergleich zu anderen Prüfungsformaten hier nicht eindeutig evaluiert werden können (Montgomery et al. 2021). Ebenso unklar ist, ob die erlernten und geprüften Kompetenzen tatsächlich in der Praxis umgesetzt werden.

Ethische Fragestellungen und Entscheidungssituationen als Thema von OSCE-Stationen sind im internationalen und fachübergreifenden Diskurs ebenfalls längst angekommen. Bereits 1993 beschreiben Singer et al. die Entwicklung und Testung zweier Prüfstationen mit ethisch-relevanten klinischen Situationen (Singer et al. 1993) und stoßen so eine Diskussion um das (richtige) Assessment ethischer Kompetenzen an (Arnold und Forrow 1993).

Durch die Konzeption komplexer Szenarien und die Einbindung von Fertigkeiten und Haltungen in das Prüfungsformat ist jedoch auch eine gezielte Überprüfung von ethisch-reflexiven Kompetenzen auf Masterniveau umsetzbar. Damit wird sowohl der Auseinandersetzung um adäquate Lern‑, Lehr- und Prüfungsformate in der Hochschulbildung, als auch der Diskussion um gewollte und unbeabsichtigte Folgen ethischen Kompetenzerwerbs Rechnung getragen.

So zeigt Kersting in einer Untersuchung zur moralischen Desensibilisierung von Auszubildenden in der Krankenpflege, dass diese verschiedene Strategien anwenden, um mit problematischen Erlebnissen in der täglichen Praxis umzugehen. Die hohe Belastung bereits in dieser Phase des Arbeitseinstiegs führt dabei zu einem Coolout. Pflegeethik ist dann „nicht Grundlage für eine gelingende Pflegepraxis, sondern das Resultat der objektiv Kälte verursachenden Bedingungen, denen sie immanent bleibt“ (Kersting 2013, S. 297). Dabei betrachtet Kersting insbesondere die pflegeethischen Ansätze von Sara T. Fry und Marianne Arndt. In ihnen sieht sie keine Hilfestellung zur Ausbildung einer reflexiven Praxis.

Auch Höhmann et al. (2016) fordern, gestaltbare Probleme der Pflegepraxis zu identifizieren und zu bearbeiten. Dabei spielen das jeweilige Kompetenzprofil und der damit verbundene fachliche Handlungsspielraum eine wesentliche Rolle. Nicht schnelle Lösungen, die losgelöst von Eigenheiten der Einrichtung und Fähigkeiten der Mitarbeiter:innen sind, sondern umfassende Änderungen auf allen Kompetenz- und Organisationsebenen müssen die Gestaltungsmöglichkeiten der Mitarbeiter:innen erweitern. Diesen Argumentationen muss sich die curriculare Entwicklung annehmen, um nicht selbst Teil einer Desensibilisierung zu werden, indem die Bedingungen unreflektiert reproduziert oder der bei Kersting aufscheinende Dualismus zwischen einer Ethik ohne Bezug zur Praxis und einer Praxis ohne ethischen Bezug manifestiert werden. Hierfür ist es notwendig, sowohl auf individualethischer Ebene als auch mit Organisations- und Gesellschaftsbezug alternative Lösungen zu kennen sowie umsetzen zu können. Dies soll mittels der Neuausrichtung des Moduls mit innovativer, praxisorientierter Prüfungsform und den entsprechenden Lehrmethoden ermöglicht werden.

Dabei stellt sich einerseits die grundsätzliche Frage, inwiefern diese Form des Lernens mittels hochschuldidaktischer Methoden gewährleistet werden kann (Wissenschaftsrat 2017). Andererseits muss die inhaltliche Passgenauigkeit und Validität des OSCE-Prüfungsformates aufgegriffen werden. Ausgehend von der Annahme, dass Wissen, Fertigkeiten und Haltungen im Rahmen der klinisch-ethischen und forschungsethischen Ausbildung vermittelt und geprüft werden sollen: Misst ein OSCE diese auch? Neben allgemeinen Fragen zur Entwicklung von Prüfungsformaten, steht hier im Mittelpunkt, inwiefern und unter welchen Bedingungen OSCEs als Prüfungsformate neben erfragbarem Wissen und beobachtbaren Fertigkeiten auch Haltungen erfassen können. In der vorliegenden Konzeption des Ethik-OSCEs soll dies über eine Operationalisierung der Haltung gelingen, d. h. dass in den Szenarien gezielt bestimmte Nachfragen oder Reaktionen herausgefordert werden, die sich aus der eingenommenen Haltung ergeben. Ergänzend wäre eine dem formativen OSCE ähnliche Reflexion denkbar, in der eine Selbsteinschätzung sowie konzeptionelle Deutung der Prüfungssituation vorgenommen wird. Diese würde jedoch nicht das Feedback der Prüfenden in den Mittelpunkt stellen, sondern die Einschätzung von Metakompetenzen, wie es Bogo et al. (2012) für den Bereich der Sozialen Arbeit beschreiben.

Arnold und Forrow schreiben 1993 zudem in ihrem Editorial zu OSCEs als Evaluationsmethode klinisch-ethischer Kompetenzen, dass diese auf die wichtigsten Dimensionen der relevanten Kompetenzen fokussieren sollten, valide Kriterien nutzen sollen, die eine klare Einschätzung ermöglichen und mit dem Verhalten der Studierenden in der Praxis korrelieren sollten. Inwiefern dieser dritte Aspekt erfüllt ist, lässt sich nur schwer beantworten und wird immer wieder in der Auseinandersetzung mit OSCEs thematisiert. Chenot und Ehrhardt beschreiben unter Bezugnahme auf Millers System zur Beurteilung von Kompetenzen, dass die „Überprüfung von Fertigkeiten in vivo“ (Chenot und Ehrhardt 2003, S. 437), also in der klinischen Praxis, auch nur dort sinnvoll ist. Für den Studiengang Pflegewissenschaft und Praxisentwicklung ist dies jedoch kaum umzusetzen. Einige Studierende arbeiten in der klinischen Praxis, andere im wissenschaftlichen Kontext. Dadurch treten organisatorische Herausforderungen zur Prüfungsumsetzung auf, die es zu bewerkstelligen gäbe. Einer Prüfung in vivo steht jedoch vor allem die Kombination der beiden Bereiche – klinische Ethik und Forschungsethik – in einer Prüfung entgegen.

Gegenstand der mit Studienstart im Wintersemester 2021 anlaufenden Evaluation ist somit zunächst die Frage, ob die Anwendung des postgraduellen „Ethik-OSCEs“ die Lernerfolge, praktischen Kompetenzen und Zufriedenheit der Studierenden beeinflusst. Dies geschieht vor dem Hintergrund der Diskussionen um Gütekriterien des OSCE-Formates, die bereits in der Entwicklung berücksichtigt wurden. So sind bspw. die Anzahl und Dauer der Prüfungssituationen auf die zu erfassenden Kompetenzen abzustimmen, sowie als wichtige Indikatoren für die Interne Konsistenz zu betrachten (Rushforth 2007; Gormley 2011). Standardisierung der Assessments und zweifache Prüfende sowie geschulte Darsteller:innen stellen wiederum Möglichkeiten zur Verbesserung der Reliabilität dar (Rushforth 2007).

Zuletzt sind, nicht nur vor dem Hintergrund der Covid-19-Pandemie, auch Fragen zu alternativen, möglicherweise digitalen Prüfungsformaten zu stellen. Insbesondere vor dem Hintergrund praktischer Lernsituationen wird immer wieder das „programmatic assessment“ thematisiert. Dieser Ansatz hat einen formativen Prüfungscharakter, aber zugleich summative Elemente, und soll das Lernen und Entscheiden in komplexen Praxissituationen unterstützen. Dabei können sich Schwierigkeiten ergeben, wenn parallel zum Lernprozess Assessments der Lernergebnisse stattfinden (Schut et al. 2021). Dennoch ist der Gedanke eines Prüfungsformates mit eher formativem Charakter grundsätzlich auch für den Bereich der Ethik denkbar. (E-)Portfolios, welche Praxiserfahrungen dokumentieren und reflektieren lassen, Lernfortschritte aufzeigen und auch eine Reflexion der Haltung ermöglichen, wären hier ein mögliches Prüfungsformat. Weitere Möglichkeiten böten die teilweise oder vollständige Digitalisierung des OSCE-Prüfformates oder Key-Feature-Assessments mittels klinisch-ethischer Fallbeispiele. Diese sind jedoch ebenso wenig in vivo Prüfungsformate wie OSCEs, fragen unter Umständen sogar lediglich prozedurales Wissen ab. So erscheint das Prüfungsformat des hier vorgestellten Ethik-OSCEs als Simulationsprüfung geeignet, die erlernten Kompetenzen zu prüfen. Die Evaluation wird hier ansetzen müssen und umfassende praktische Fragen zur Implementierung, zu Auswirkungen auf Methoden und Qualität der Lehre, aber auch grundsätzliche Fragen der bereits angesprochenen Passgenauigkeit des Prüfungsformates und der Anschlussfähigkeit an tatsächlich auftretende praktische Herausforderungen sowie erforderte Kompetenzen thematisieren.
